# Making better decisions in groups

**DOI:** 10.1098/rsos.170193

**Published:** 2017-08-16

**Authors:** Dan Bang, Chris D. Frith

**Affiliations:** 1 Wellcome Trust Centre for Neuroimaging, University College London, London WC1N 3BG, UK; 2 Interacting Minds Centre, Aarhus University, 8000 Aarhus, Denmark; 3 Institute of Philosophy, University of London, London WC1E 7HU, UK

**Keywords:** decision-making, social, bias, confidence, diversity, Bayesian

## Abstract

We review the literature to identify common problems of decision-making in individuals and groups. We are guided by a Bayesian framework to explain the interplay between past experience and new evidence, and the problem of exploring the space of hypotheses about all the possible states that the world could be in and all the possible actions that one could take. There are strong biases, hidden from awareness, that enter into these psychological processes. While biases increase the efficiency of information processing, they often do not lead to the most appropriate action. We highlight the advantages of group decision-making in overcoming biases and searching the hypothesis space for good models of the world and good solutions to problems. Diversity of group members can facilitate these achievements, but diverse groups also face their own problems. We discuss means of managing these pitfalls and make some recommendations on how to make better group decisions.

## 1. Why is it hard to make decisions?

Most decisions have to be made in the face of uncertainty and in the absence of immediate feedback. Making decisions in groups can reduce uncertainty, and this is one of the reasons why it is observed frequently throughout the animal kingdom [1,2]. For example, a shoal of fish can follow a light-level gradient that is too weak a signal for an individual fish to follow [3]. Humans can develop better models of how the world works by means of discussion [4,5]. However, decision-making in groups is complex and can go wrong [6,7]. The purpose of this paper is to review the scientific literature in order to identify pitfalls that decision-makers—both individuals and those making decisions in groups—should be aware of and to make recommendations that can help groups make better decisions.

Our review will mostly be concerned with small groups who agree on the problem to be solved, such as panels and committees, although many of the phenomena that we consider can also be observed in large groups. We adopt a Bayesian framework which has been shown to capture many aspects of intuitive decision-making [8–10]. The term *intuitive* is important; it reminds us that we are not conscious of most of our cognitive processes, which happen automatically and are simply too fast to reach awareness. We will often refer to the Bayesian distinction between *past experience* (prior) and *new evidence* (likelihood). We will also refer to the need to explore the hypothesis space from which we select an action. In doing so, our main aim is to understand how decisions can go wrong. Details of the Bayesian approach can be found in appendix A.

## 2. Introducing some basic concepts

### 2.1. Forming beliefs in an uncertain world

#### 2.1.1. Weighting past experience and new evidence

It is important to strike the right balance between, on one hand, past experience and perceived wisdom and, on the other hand, new evidence. In the middle of the last century, doctors sent large numbers of children to hospital to have their tonsils and adenoids removed. Such referrals were made even though, in 1938, the Schools Epidemic Committee of the Medical Research Council concluded: ‘It is a little difficult to believe that among the mass of tonsillectomies performed to-day all subjects for operation are selected with true discrimination and one cannot avoid the conclusion that there is a tendency for the operation to be performed as a routine prophylactic ritual for no particular reason and with no particular result’ [11]. In a more recent empirical study, it was found that, in 1981, 17% of doctors used coronary angiography inappropriately; they did not keep up with the evidence and stuck with apparently tried and trusted experience [12,13].

#### 2.1.2. Assessing the reliability of our sources

Even when we make decisions on our own, information often comes from other people. To use this information appropriately, we need an estimate of the *reliability*, known as *precision* in the Bayesian framework, of our sources. The confidence with which others transmit information can be a useful marker, but it can also be misleading, even when there is no intention to deceive. These dangers are present even when evaluating our own judgements. In many situations, the confidence we feel might not be a good guide. For example, a victim of a crime may sincerely believe that they have a good memory for the face of the criminal, but select an innocent person in an identity parade [14].

### 2.2. Finding the best solution

#### 2.2.1. Sampling the hypothesis space

**Figure 1.**
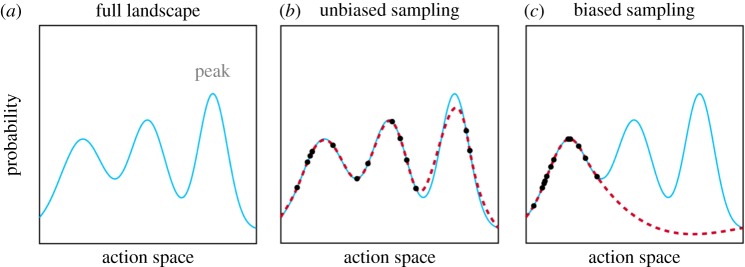
Exploring the landscape of possible actions. (*a*) The plot shows the probability distribution over the goodness of possible actions. The peak indicates the best action. (*b*) A rough estimate of the probability distribution can be made by drawing samples. (*c*) If the sampling is biased, then the estimate of the probability distribution may not reflect the true one. To create the sample-based distributions, we drew samples from the true probability distribution (*N* = 14) in a uniform manner (*b*) or from a sub-part (*c*), and then applied a smooth function. We sampled the height of the true probability distribution, akin to remembering how good an action was or asking a friend for their advice about which action to take.

We can think of the task of choosing the best action as one of finding the highest point in a hilly landscape [15] (figure 1*a*). The landscape represents a probability distribution over the goodness of possible actions where the highest probability indicates the best action. But how can we find this peak? A calculation of the entire distribution is often computationally intractable, and yet there are many circumstances in which the brain achieves near-optimal solutions [16,17]. One way this might be achieved is by *sampling* the probability distribution [15]. By visiting the landscape at different points, we can form a rough map and thus make a better choice (figure 1*b*). For example, we may search our memory for situations similar to the current one [18,19] or ask others for advice [20].

Sampling works well in domains such as perception and motor control where we can draw on a rich database built through years of experience [21,22]. Its disadvantage is, however, revealed by the errors that people make in unfamiliar abstract settings; we should engage in unbiased sampling, but, instead, we fall prey to old habits [23]. Another risk of sampling is that the explorer of the landscape risks getting stuck on a local peak and thus never discovers that there is a higher peak (figure 1*c*). Having found such an apparently satisfying solution, people tend to devote their cognitive resources to justifying it, rather than looking for a better one [24].

#### 2.2.2. Exploitation and exploration

When we think we know enough, or are running out of time, we *exploit* our current knowledge to choose the action that we think will achieve the best outcome. We would normally prefer an outcome of high value, but we also take account of the probability that an outcome will be realized at all [25]. However, as shown by the success of lotteries [26], estimating the *expected value* of an action is subject to strong biases. There are many cases where we do not have sufficient knowledge to make a good decision. In such cases, we should *explore* rather than *exploit* and seek more information before making up our minds [27,28]. Of course, if we have an exaggerated opinion of the adequacy of our current knowledge, then we may fail to seek more information. We have already mentioned the observation that some doctors used coronary angiography inappropriately [12,13]. If they had collected exercise data they would have found that angiography was unnecessary.

### 2.3. Hidden biases

In the interest of processing information efficiently, humans employ shortcuts, many of which evolved by leading to life-saving actions in dire situations [29]. For example, rapidly recognizing an enemy, or a predator, leads to the good decision to take evasive action. However, in modern life, these biases, left over from evolution, can cause poor decisions; for example, rejecting a candidate because they are from an ‘unfamiliar’ group (e.g. because of ethnicity or gender) and trigger a threat response [30]. As modern-day humans, we are surprised at these ‘outdated’ biases when they are pointed out and strive to be free of them [31]. There are other kinds of biases too, which depend on individual experience. For example, one can imagine a culturally dependent bias to stand on the left side of escalators (e.g. Tokyo). While a useful instinct when in the context in which the bias was learnt, the bias can be offensive when in a new context where it is customary to stand on the right (e.g. Osaka or London).

## 3. When individual decisions go wrong

We will now consider some of the ways in which individual decisions can go wrong, and then discuss how groups can, sometimes, overcome these shortcomings. We will mainly consider decisions where we are aware of the problem and the answer that we reached but where we need not be aware of how we got there—even when we do think we know, we might be far off the truth [32]. This mode of decision-making is the most typical of the workings of small groups.

### 3.1. Forming wrong beliefs

#### 3.1.1. Too much or too little faith in past experience

A common source of bad decisions is inappropriate prior beliefs. If we have a strong prior belief in a hypothesis, then we need huge amounts of conflicting evidence to change our mind. For example, the surgeons performing tonsillectomies in the 1940s had the strong belief that this operation would be of benefit for all and were not swayed by the report from the Medical Research Council [11]. In physics, Einstein's firm belief that the universe was static led him to add an unnecessary parameter (the cosmological constant: Λ) when he applied his theory of general relativity to the universe [33]. In geology, the theory of continental drift was rejected for 40 years because of ‘prior theoretical commitments' to permanence theory [34]. Conversely, if we have a weak prior belief in a hypothesis, then we need huge amounts of supporting observations to believe it. For example, most scientists do not believe in extra-sensory perception (e.g. telepathy) [35]. As a result, they demand much stronger evidence for the existence of extra-sensory perception than for more widely accepted hypotheses [36]. While perhaps sensible in the case of extra-sensory perception, such weak prior beliefs have hindered scientific advances in the past.

#### 3.1.2. Misinterpreting new evidence

**Figure 2.**
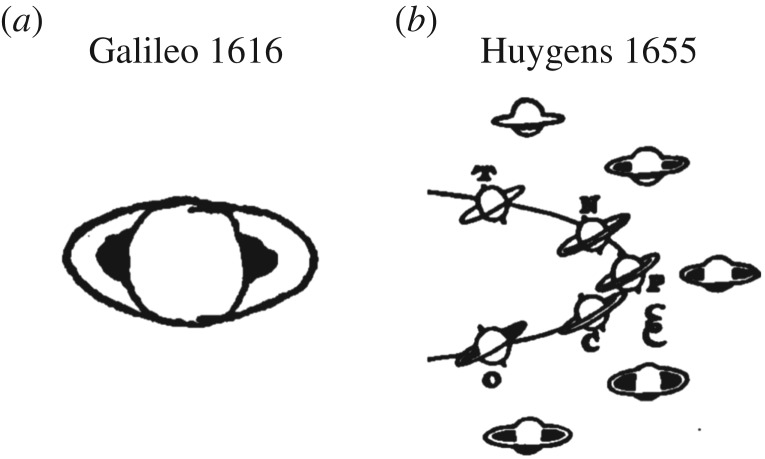
Misinterpreting new evidence. (*a*) Galileo was mystified by the appearance of Saturn, which changed over time and appeared to have ears or arms. *Le Opere di Galileo Galilei, XII, Correspondence 1614–1619, Letter 1223.* (*b*) Huygens recognized how these changing appearances could be explained by a ring. *Systema Saturnium* (1659), *Oeuvres Completes de Christiaan Huygens*, XV: 312.

It is not the case that faith in prior beliefs is a bad thing. Prior beliefs reflect past experiences with the world, either on an evolutionary or an individual time scale, and we greatly benefit from these. It is also not the case that new evidence should be distrusted on principle, because it is imperative that we adapt to new situations. However, if we are to update our beliefs about the world appropriately, we need to be able to interpret the new evidence correctly. Here is an example from the history of science. When Galileo first viewed Saturn through a telescope in 1610, he did not have a good model to explain what he saw. At one point, he described the planet as having ears (figure 2). It was not until 1655 that Huygens realized that these ‘ears’ were the rings that surround Saturn [37].

#### 3.1.3. Erroneous evaluation of rare events

When updating our beliefs about the world, whether consciously or unconsciously, we seem to be especially bad at dealing with rare events; we overestimate their occurrence when our beliefs about the world are based on information obtained from others, but underestimate them when they are built from our own experience [38,39]. For example, after reading the leaflet for a prescribed medicine, we might overestimate the probability that a physical symptom is due to an adverse side effect. By contrast, a doctor with years of experience prescribing the medicine might underestimate that very same probability. In this example, the patient overweights the new evidence, whereas the doctor overweights their past experience.

#### 3.1.4. Too much or too little faith in one's own abilities

Most people, whether they like it or not, are bad at gauging the accuracy of their beliefs [40]. In a typical psychological study, people would be asked to indicate their confidence in different judgements (e.g. that a cloud of dots is moving to the left and not to the right) or propositions (e.g. that Rio de Janeiro is the capital of Brazil) as a probability of being correct [41]. The researcher would then quantify two aspects of the relationship between people's confidence and beliefs [42]. The first one is *resolution* which characterizes the extent to which people's low and high confidence can discriminate between their incorrect and correct beliefs. The second aspect is calibration which characterizes the extent to which their stated probability of being correct reflects their objective probability of being correct; for example, when they say that there is a 70% chance that their belief is correct, then they are also correct 70% of the time. Calibration in particular is subject to biases. People are often overconfident for hard problems, but paradoxically, they tend to be underconfident for easy ones—a phenomenon known as the *hard*-*easy effect* [43–45]. There are, however, significant individual differences in the degree to which people display under- or over-confidence [46].

### 3.2. Simple solutions to complex problems and their unforeseen consequences

#### 3.2.1. The Streisand effect

Many problems require a depth of thinking that is beyond our cognitive powers. Even a simple game like tic-tac-toe can unfold in thousands of different ways. When faced with problems of high complexity, we tend to resort to *heuristic* strategies—that is, simple algorithms, or rules of thumb, for selecting an action [47]. Heuristic strategies can save us time and cognitive effort [48], but they might also have unintended consequences. One such consequence has become known as the *Streisand effect* [49]. In 2003, Barbra Streisand filed a lawsuit to prevent the online posting of a photo of her home. At first sight, this seems to be the appropriate way to prevent unwanted material being made public. We believe that if unwanted behaviour is punished then it will cease. Prior to the lawsuit, only six people had downloaded the photo, two of them being Streisand's lawyers. After the attention created by the lawsuit, about 400 000 people visited the website [50].

#### 3.2.2. Learning without a model of the world

One heuristic solution to the complexity problem is to use *model-free­* strategies instead of *model-based* strategies [51], which are slow to develop [52] and cognitively taxing [53]. Model-free strategies proceed by storing the outcomes of past actions and then acting upon these values in a habitual manner. For example, a model-free player of tic-tac-toe might always seek to occupy the centre of the grid, because such behaviour has been rewarded in the past. By contrast, model-based strategies proceed by building and updating a model of the world; a model-based player of tic-tac-toe would not rely on old habits, but draw on an internal model of their opponent, imagining and assessing their future moves. As should be apparent, for model-free strategies to work well, substantial experience in an unchanging world is needed [51]. This requirement is, however, rarely satisfied. Even the state of the decision-maker may change, such that the future state to which the decision is relevant is not the same as the state when the decision had to be made. As we all know, if we shop when we are hungry, we are likely to make decisions that our future satiated self would disapprove of [54].

#### 3.2.3. Too few hypotheses

Another heuristic solution to the complexity problem is to consider only a subset of hypotheses about the world and possible actions. This strategy is particularly likely to be applied to problems where our past experience is not rich enough to guide sampling of the full space of hypotheses in an adequate manner [15]. Reduction of this space may, however, lead to bad decisions when too few alternatives are taken into account [55]. For example, the political scientist Philip Tetlock divided political pundits into *foxes*, who consider multiple hypotheses, and *hedgehogs*, who consider much fewer; in general, the foxes are more accurate in their predictions, but the media prefer to hear from the hedgehogs [56].

#### 3.2.4. Inertia and optimism bias

The last solution to the complexity problem that we will consider is sampling from memory: instead of using forward planning, imagining and evaluating the future consequences of an action, we may decide on an action by recalling what we did the last time that we were in a similar situation [18,19]. This strategy can explain the observation that people tend to repeat past choices regardless of the current evidence—a bias known as *decision inertia* [57]. When we do engage in forward planning, we may still use sampling from memory to inform some of the computations, such as estimating the expected value of an action. This strategy may, however, lead to distorted estimates if the sampling is biased. For example, it has been shown that our belief about the probability of an action outcome depends on how *desirable* we find that outcome—a phenomenon known as *optimism bias* [58]. This bias may come about if, when building expectations from memory, we sample outcomes we like, but ignore outcomes we do not like. As a result, we may underestimate the probability of undesirable outcomes, such as illness resulting from smoking, and overestimate the probability of desirable outcomes, such as winning the lottery or our new restaurant being a hit. Interestingly, when gathering new information, we seem to prefer sources which give us good news, which only biases our memory further [59].

## 4. The advantages of decision-making in groups

Many of the problems of individual decision-making can be mitigated if individuals join with others to make decisions in a group. We will consider group scenarios where people work together or independently. We will not discuss the nature and function of group leaders as this is a field in its own right [60–62]. However, in the Recommendations section, we will mention some situations in which a group chair or leader can help mitigate the problems specific to group decision-making.

### 4.1. Forming better beliefs

#### 4.1.1. Benefits of pooling information

As a statistical rule of thumb, pooling information across independent individuals leads to more reliable information [63,64]. For example, pooling unbiased but noisy numerical estimates causes uncorrelated errors to cancel out and therefore increases the precision of the pooled estimate (see appendix B1). Here, estimation errors may be uncorrelated, because people base their estimates on different past experiences or new evidence. The benefit of pooling information across individuals was first shown by Francis Galton [65]. He collected together the individual entries for a ‘guess the weight of the ox’ competition and showed that the average of these entries was closer to the truth than the single winning entry. This effect has been replicated experimentally for small groups [4,66] and large groups [1,67].

#### 4.1.2. Wisdom of crowds

The promise of pooling information underpins recent attempts to harness the *wisdom of crowds* [68]. Central to these attempts has been the development of methods for combining individual judgements in cases where it is hard to establish people's expertise or honesty, such as when information is elicited over the Internet [69,70]. For some domains, the method used need not be complex. For example, a recent set of studies showed that, by adopting the decision favoured by the majority of independent dermatologists, the accuracy of skin and breast cancer diagnosis can be improved over and above the single-best individual [71–73]. This approach to diagnosis can overcome some of the issues revealed by advocates of evidence-based medicine [12].

#### 4.1.3. Majority decisions

**Figure 3.**
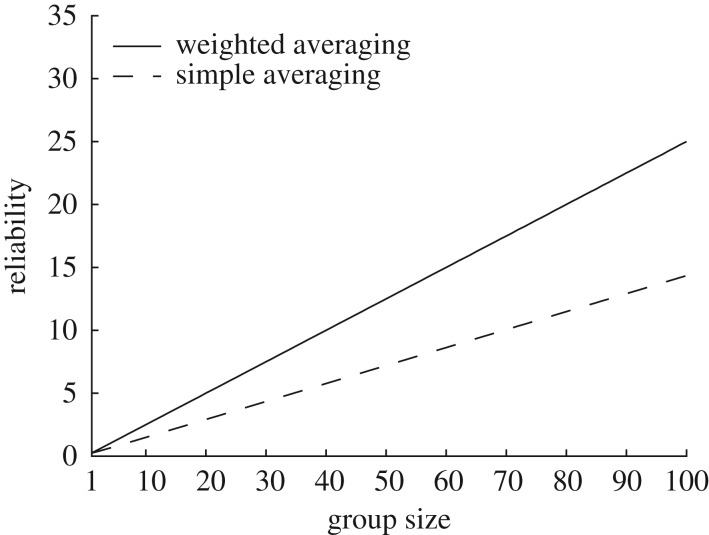
Weighting by reliability. The figure shows that the reliability of a pooled estimate is higher when each individual estimate is weighted by its reliability (weighted averaging) than when assigning equal weights to all individual estimates (simple averaging). In this simulation, we assumed that the individual estimates varied in terms of their reliability and were uncorrelated. See appendix B2 for mathematical details.

A common strategy for combining individual opinions into a group decision is to let each member vote on the available options and select the option favoured by the majority [74,75]. This *majority rule* may be perceived as the fairest solution if group members have very different preferences. However, the outcome of this process critically depends on the reliability of the information upon which individual opinions were based. It is therefore often advisable to use a *weighted* majority rule where individual reliability is taken into account (figure 3). But how should reliability be assessed?

#### 4.1.4. Social markers of reliability

One marker of reliability is *status* [76,77]. Group members may enjoy high status because of their rank (pay grade), seniority (time in office) or experience (he has been to war)—traits which we view as markers of reliability [78–80]. Another marker of reliability is the *confidence* with which an opinion is expressed [81–84]. Group members may express high confidence, because they have relevant past experience (prior) or strong evidence (likelihood) [41]. One solution to the weighting problem may be to ask each individual for an estimate of the proportion of people that they think will share their opinion: intriguingly, it can be shown that a procedure which assigns higher weight to opinions that are more popular than predicted generates better decisions than either the majority rule or the confidence-weighted alternative [70].

#### 4.1.5. The remarkable success of open discussion

Discussion among members of small groups, when there is no time pressure, has proved an excellent strategy for making good use of the knowledge held by group members [85–91]. The reason revealed in these studies is that discussion involves a *recalibration* of markers of reliability. By arguing for or against the opinions put forward, we can assess the evidence on which each opinion was based [24]. In general, we are more likely to be swayed by a well-argued opinion than an opinion that is merely stated with high confidence [91]. By means of such recalibration, we can together increase the probability that no opinion is assigned undue weight.

### 4.2. Finding better solutions

#### 4.2.1. Pooling cognitive resources

Groups have been shown to outperform individuals for many problems of probability and reasoning [92,93]. For example, in the *Wason selection task*, a well-known problem of logic, only 10–20% of individuals give the correct answer, but this increases for groups to around 70%. Groups also outperform individuals in economic games (e.g. *Beauty-Contest*) [94]; find more efficient solutions to numerical problems (e.g. calculating tax returns) [83,95] and reach a level of abstraction for scientific topics (e.g. the concept of biological transmission) that is higher than expected from the sum of the members working alone [96]. Importantly, the benefits of having worked in a group can transfer to individual contexts, with individuals retaining good strategies developed together with others [83,92].

#### 4.2.2. Combining efforts of explorers and exploiters

We can distinguish between people who tend to be *exploiters* and those who tend to be *explorers* [97]*.* Exploiters prefer to stay with their current model of the world, rather than switch to another. They consider a small part of the hypothesis space, refining the solution that first came to mind. Explorers, in contrast, prefer breadth. They consider a much larger part of the hypothesis space and are therefore less likely to be trapped on a local maximum. Their exploration activity, on the other hand, means that they may decide to act when it is too late [27]. The extent to which people exploit or explore is in part a matter of personality and of genetics [98]. Many animal groups, from honeybees to humans, contain a mixture of exploiters and explorers. A typical swarm of 10 000 honeybees will contain between 300 and 500 scout bees [99]. A mixture of such diverse individuals can create advantages for the group.

### 4.3. Overcoming hidden biases

Groups can help us discover the ‘beam that is in thine own eye’ (Matthew 7:3, KJV). While our own biases are often hidden from ourselves, we are remarkably good at detecting others' biases [100,101]. Another way in which groups can help individuals overcome individual biases is by changing the incentive structure of the problem at hand, either indirectly (e.g. reputation loss or gain) or directly (e.g. financial loss or gain). In some tasks, for example, group members spontaneously perform better than they would have had they been doing the task alone [102,103]. This enhancement, known as the *Köhler effect* [104], is thought to arise because group members do not want to be perceived as the weakest link [103,105]. When providing financial incentives, it is critical to strike a balance between individual incentives, which are distributed unevenly within the group, and group incentives, which are distributed evenly [106]: while individual incentives improve the speed of group decisions, group incentives improve accuracy [107]. Decisions, however, often cannot be both fast and accurate [108].

## 5. When group advantages are dissipated

Groups can overcome some, but not all, of the problems of individual decision-making. We will now consider a number of potential pitfalls facing group decisions, such as lack of independent knowledge, biases that skew the sharing of information or preferences and the problem of competing individual and group goals.

### 5.1. Lack of independent knowledge

**Figure 4.**
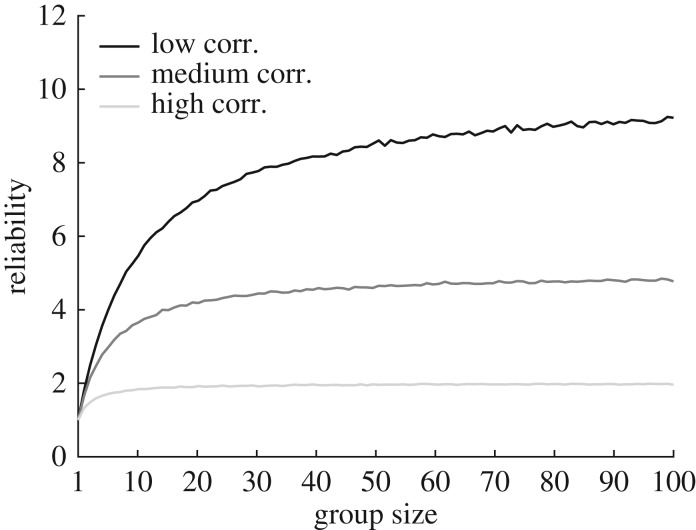
Information-limiting correlations. The figure shows that the reliability of a pooled estimate saturates when the pooled information is correlated. In this simulation, we assumed that the individual estimates were equally reliable and correlated to a low, medium or high degree. See appendix B3 for mathematical details.

*Groupthink* is perhaps the most well-known cause of bad group decisions. The term, which was popularized by the psychologist Irvin Janis in his case study of the Bays of Pigs Invasion [7], has been used with many meanings, but a common theme is that group members become less independent and behave as if they were a supersized individual. There are largely two causes of a lack of independence. First, group members are too similar to each other: their knowledge is based on similar past experiences and/or similar new evidence. Second, group members, even when initially dissimilar, adapt to each other's knowledge through social interaction. As a consequence of such correlations, individual errors are no longer independent and the benefit of pooling information across group members is reduced (figure 4).

#### 5.1.1. Group members are too similar to each other

It is often unavoidable that members of a group have had similar past experiences and therefore hold similar expectations about the world. In our social life, we tend to get together with people with whom we share backgrounds and personal characteristics [109]. In the workplace, we are often paired with people who have taken a path in life not too different from our own [109]. This tendency for ‘birds of a feather to flock together’ can increase group cohesion, but it can also have a negative effect on group decisions*.* When group members hold similar prior beliefs, their inferences will be biased in similar ways. This problem cannot be alleviated by asking group members to work independently. For example, members of the same political party are likely to interpret incoming data in the same way regardless of whether they discuss their interpretation with each other.

It is also often unavoidable that members of a group acquire similar information about the world. Similar people tend not only to have similar past experiences but also to acquire new evidence in similar ways. For example, they may read the same newspapers or listen to the same radio programmes. This problem is amplified on the Internet where search engines (e.g. Google) and social media (e.g. Facebook) organize content according to our location and browsing history—promoting information that fits our preferences, those of our friends or people who are estimated to be like us [110]. This personalization facilitates the creation of *filter bubbles* [111] and *echo chambers* [112] where information is created and recycled by like-minded individuals. When group members obtain correlated evidence, their conclusions will covary—regardless of whether they have correlated past experiences [113].

#### 5.1.2. Group members converge too strongly

We have considered how individuals can hold non-independent knowledge because of circumstantial factors. Individuals may, however, actively adapt to each other's knowledge through group interactions—a phenomenon studied under the headings of *herding*, *conformity* and *social influence* [114–116]. Here, we distinguish between two types of social influence: people may adapt to each other's knowledge because of a desire to fit into the group or through believing that others have better knowledge.

#### 5.1.3. Desire to fit into the group

A classic example of social compliance is Salomon Asch's line-judgement experiment [117]. Groups of eight male students were shown lines of varying length and asked to announce in turn which one matched a reference line. All but one of the participants were actors, instructed by Asch to give the wrong answer. Surprisingly, participants, who were seated such that they would always announce their answer last, yielded to the majority about 40% of the time, despite privately knowing the correct answer. More everyday examples include not admitting to our highbrow friends that we prefer milk chocolate and sweet wine.

#### 5.1.4. Believing that others have better knowledge

In uncertain situations, individuals can gain information by observing others. When we are ignorant or learning is too tedious, we do well to ‘copy the majority’ or ‘copy the most successful’. These shortcuts capitalize on the facts that behaviours tend to be popular for a reason and that people tend to be successful because their behaviour is adaptive [20]. When we ourselves have useful information, these shortcuts may seem less useful. Still, we often ignore our own instincts and follow others instead [118,119]. For example, we may join a long queue of shoppers at a market stall because we assume that others are shopping there for a good reason. We may do this even if we believe that the goods at the stall are of dubious provenance. The psychological assumption that others typically act on good information can explain why beliefs and behaviours can rapidly spread through a group—in a so-called *information cascade* [119]—and why rational agents can make decisions that go against their own better knowledge.

#### 5.1.5. Information cascade

Financial bubbles are an instructive example of an information cascade. A financial bubble is typically defined as the trading of an asset at a price that is much higher than its intrinsic, or true, value. It is, however, important to bear in mind that financial bubbles are identified in retrospect, once there has been a sudden drop in the price of the asset. During the build-up of a financial bubble, an individual trader may be uncertain about the true value of the asset and therefore be justified in inferring that buyers of the asset are acting on good evidence. For example, the trader may believe that the buyers have inside information that the asset is about to increase in value. In a sense, the trader is behaving as if the market is an agent with beliefs and intentions [120]. In this way, small fluctuations in trading activity can end up having huge financial implications.

### 5.2. Hidden group biases

#### 5.2.1. Shared information bias

One well-established finding in the scientific literature is that group discussions tend to focus on information that is shared by all group members, often at the expense of information that is essential but only held by a minority [121]. This phenomenon is known as *shared information bias* or the *hidden-profile effect*. For example, a fellowship or a grant panel, which is often made up of people with different areas of expertise, may focus on factors that everybody is familiar with, such as an applicant's track record, and pay less attention to the parts which require specialist knowledge, such as the risk involved in using a new method [122]. As a result, the success of an application may depend more on the composition of the reviewing panel than the quality of the proposed work. There are a number of reasons for this focus on shared information. Circumstantial factors play a role. Shared information is more likely to be sampled, because it is held by a greater number of people [123]. The need to make fast decisions makes it less likely that important but time-consuming information is discussed [124]. Psychological factors also play a role. Group members may focus on shared information to increase their standing in the group as others tend to like us more, and judge us as more competent, if we know what they already know [125].

#### 5.2.2. Amplifying biases

Groups often amplify the initial preference held by the majority of its members—an effect known as *group polarization* [126,127]. For example, when the initial preference of the majority is risk-seeking, then the group may take on more risk than its members would have done on their own [128]. By contrast, when the initial preference of the majority is risk-averse, then the group may make more cautious choices than its members would have done on their own [129]. Group polarization has been shown in high-stake situations, including courts of law. Here, jury members tend to shift towards harsher or more lenient verdicts after deliberation [130], and groups of judges take more extreme courses of action [131] than would have been expected given their initial preference.

There are different theories about this phenomenon [127,132]. One explanation is that preferences are spread by contagion, similar to moods [133] and automatic behaviours [134]. Evidence shows that the way in which our brain represents expected value adapts to relevant others; for example, a usually patient person, who interacts with an impatient other, comes to prefer immediate gratification [135,136]. Another factor is uncertainty about our own preferences [137], which makes us look to others for cues [138]. If we hear new arguments in favour of an initially weak preference, then it may make sense to strengthen it.

### 5.3. Competing goals

#### 5.3.1. Status and accountability

Because humans have many complex and competing goals, it is only to be expected that some of us are more concerned with our *status* and *reputation* in the group than with making a good decision [139]. These motives can have disastrous consequences. Examples that have been prominent in the media are plane crashes and surgical errors where junior individuals did not voice valid concerns or were ignored by more senior colleagues [140,141]. A related factor which can become a preoccupation for group members is *accountability*—that is, the expectation that they will be asked to justify their opinions or actions to the group [142,143]. Group members may go to great lengths to lessen accountability. For example, they may shift their opinion towards the majority view regardless of their initial position. In contrast, when they are constrained by past commitments, they often spend their time justifying their initial position—a phenomenon known as *defensive bolstering* [143]. While the former behaviour may lead to the suppression of valid but minority viewpoints, the latter may waste valuable time and resources.

#### 5.3.2. Social loafing

In many cases, individuals can enjoy the fruits of others' labour, exerting no or minimal effort themselves. This problem, known as *social loafing* or *free-riding*, occurs when group members can receive an equal share of a group benefit regardless of individual contribution (e.g. free healthcare funded by taxes or a shared grade for a school project). As a result, the group benefit may be depleted too soon, or not be as great as it could have been had everyone given their best. The classic example of social loafing is rope pulling where groups of individuals exert less effort than when they pull the rope individually—a relationship which only grows with group size (the Ringelmann effect; [144]). Several factors promote social loafing [145]: the belief that one's contribution cannot be identified, the belief that one's contribution is dispensable and the expectation that others will free-ride on one's contribution. Some people are more likely to hold these beliefs. For example, males [145], individuals from Western societies [145] and individuals who view themselves as better than average [146,147] are more likely to engage in social loafing, possibly because they have an inflated sense of the extent to which others will benefit ‘unfairly’ from their contribution.

## 6. Diversity as a means of recovering group advantages

A solution to the problem of group members becoming too similar to each other is to increase the diversity of the group.

### 6.1. Identity and functional diversity

We can distinguish between *identity* diversity, which refers to differences in personal characteristics such as gender, age and cultural background, and *functional* diversity, which refers to differences in how people cognitively represent and solve problems [148]. Identity diversity stimulates individual thought; people who are not like us make us reconsider our own position [149,150], reducing the risk of being stuck on a local peak when a better solution exists [151]. Functional diversity, which can be facilitated by identity diversity [152,153], ensures that the group more thoroughly searches the hypothesis space for better models of the world and better solutions to the problem at hand [148].

### 6.2. Cognitive division of labour

One common strategy for increasing functional diversity in a group is to assign each individual a unique role or area of expertise [154]. For example, in businesses, co-workers often develop, spontaneously or deliberately, *transactive memory systems* through which they encode, store and retrieve knowledge [155,156]. In particular, in the encoding stage, group members identify each other's areas of expertise. In the storage stage, information is passed on to the group member with the relevant expertise, which ensures fast individual learning with minimal effort. In the retrieval stage, a group member wishing to obtain certain information can simply turn to the established expert. If the information turns out to be unreliable, then the assignment of areas of expertise is revised, and the weight of the information source is adjusted. A transactive memory system thus allows groups of individuals to divide the search through the space of hypotheses and compare solutions refined by experts. The effectiveness of the system can be improved by appointing a ‘meta-knowledge’ champion who is aware of everyone's expertise and functions as a catalyst for information exchange [157].

## 7. Problems created by diverse groups

We can think of a group of people with different areas of expertise as a *super-brain* [158]. The group members correspond to populations of neurons which perform different functions, but whose output is brought together to make sense of the world [159]. There is, however, more room for error in groups than in the brain. The brain has already solved the problem of competition for influence [160] and relies on a central executive system to coordinate information processing [161]. Having a diverse group of people may result in time-consuming processes that in the end may not avoid miscommunication [152,153].

### 7.1. Inappropriate assessment of reliability and confidence

**Figure 5.**
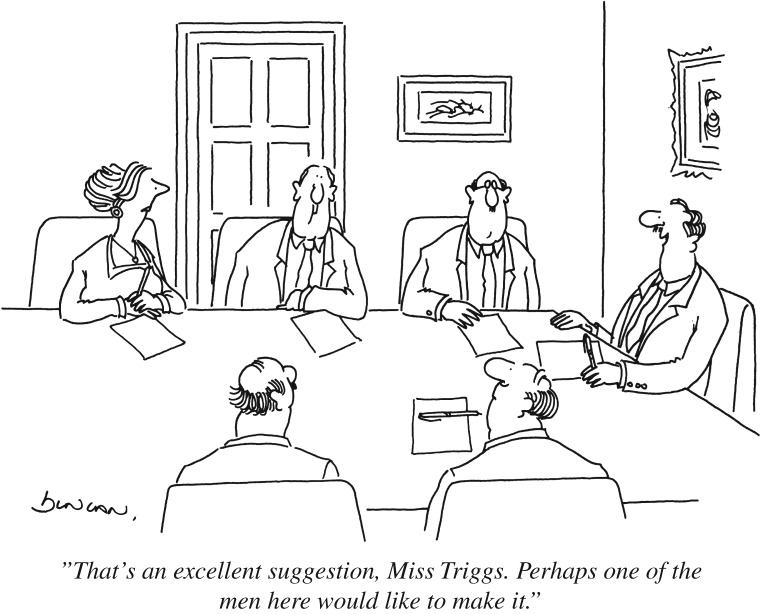
The influence of an opinion on group decisions sometimes does not depend on how good it is but on who voiced it.

We have seen how groups can make better decisions by weighting each opinion by its reliability (figure 3). It is, however, hard to judge the reliability of others' opinions if they are different from ourselves. In these cases, we often resort to inappropriate shortcuts. For example, we may view someone who is able to talk for a long time about a topic as an expert, but, as we all know, the most talkative people are not always right [162,163]. Even among highly educated people, women tend to be implicitly perceived as less competent than men [164]. This hidden bias can lead to the opinion of a women being ignored until it is voiced again by a man (figure 5).

Another reason why it can be hard to judge the reliability of others’ opinions is that markers of reliability reflect many different factors. Take confidence as an example. There is an interesting and possibly surprising link between a person's status and their readiness to take risks confidently [165]. When our status is high, we have a lot to lose when proved wrong, and this makes us more cautious. However, when our status is low, we can be bold, as we have everything to gain if we turn out to be correct. Furthermore, there is often an ambiguous link between ability and confidence. Ironically, less competent individuals tend to have an inflated sense of their own ability [166]. There are also substantial individual differences in overt confidence, which vary with individual characteristics, such as personality [167], gender [168] and culture [169]. This variation increases the risk of miscommunication among diverse group members. The complexity brought out by diverse groups is perhaps one of the reasons why we tend to feel more comfortable in groups of individuals with similar background and cultural identity [109,170,171].

### 7.2. Equality bias

So far, we have not touched on genuine differences in ability, but of course they exist and, to come to a good decision, it is often necessary to discard, or at least discount, the opinions of less competent group members. This turns out to be difficult for us to do. Studies have shown that there is a bias in small groups to treat everyone as equal in terms of ability and thus assign an equal weight to each opinion [172]. In addition, members of small groups have been shown to match each other's overt confidence, further blurring differences in ability [173]. There are various possible explanations of such *equality bias*; perhaps it serves to avoid conflict [174] or to diffuse the responsibility for difficult decisions [175]. The consequence of the bias is that, if a poor solution to a task is offered by an incompetent member and responses are pooled, this will inevitably drag down the accuracy of the group decision. When attempting to increase group diversity, we need to pay close attention to differences in ability. This is a tall order, as ability can be hard to assess in the absence of prior interactions or immediate feedback.

## 8. Recommendations

Our discussion of the literature builds on our interest in how groups of individuals make sense of information about the world, and how research can inform real-world decision-making. The findings that we have discussed are especially relevant to the workings of small groups, such as panels and committees, that make appointments and award grants. Such groups are committed to making good decisions and strive to make even better decisions. Many of the issues we have covered will merely seem good sense and have already been adopted in practice (e.g. at the Royal Society [172]). But, as is so often the case, it is easier to spot good sense in hindsight. With this in mind, what recommendations can we give?

### 8.1. Harnessing diversity

We have seen that having a diverse group of people facilitates the search for good models of the world and good solutions to problems [176,177]. There is, however, no guarantee that diverse groups will make better decisions than homogeneous groups. Sometimes, diversity leads to conflict, and no decision can be made at all. Sometimes, it causes miscommunication about who is more likely to be correct and the wrong action is taken. We have a ‘Goldilocks’ situation: individuals who differ too much can be as bad as individuals who are too similar. To harness the benefits of diversity, we must manage it appropriately.

#### 8.1.1. Recruiting diversity

There is evidence for bringing together individuals who differ in terms of their identity (e.g. gender, age or culture), cognitive style (e.g. explore and exploit) and preferences (e.g. desires and goals). First, diversity in identity reduces the harmful effects of correlated past experiences and evidence: diverse people will tend to draw on different experiences and gather information in different ways. Second, diversity in cognitive style ensures a wider coverage of the hypothesis space over possible states of the world and possible actions that one could take [176]. Finally, diversity in preferences can decrease group polarization; when there is no single preference that is favoured by the majority of the group, individual differences smooth out rather than amplify [178,179].

#### 8.1.2. Fostering diversity

Diversity may be built into the architecture of a group through specialization; specialization decreases the overlap of group members' knowledge, solutions and preferences [154]. One way to achieve specialization in an ad hoc manner is to divide the current task into sub-tasks. Offering individual incentives for completion of a sub-task, or holding individuals directly accountable for a sub-task, can facilitate specialization [180]. One advantage of increased identifiability of individual roles is that it reduces social loafing [181,182], perhaps because of the prospect of negative evaluation [183]. However, increased accountability can have unwanted side effects, such as group members suppressing original ideas for fear of negative evaluation [184], or wasting time and resources trying to defend exactly why they did as they did [143].

#### 8.1.3. Avoiding miscommunication

If group members do not have a shared frame of reference, this can make information exchange inefficient. For example, when a grant panel scores applications, panellists from different backgrounds may give a different meaning to the grades on the scoring scale. In this case, tallying the grades can be very misleading [185–188]. Another example of this communication problem comes from the world of geopolitical forecasting which deals in statements of uncertainty. In the CIA note NIE 29–51, ‘Probability of an Invasion of Yugoslavia in 1951’, Sherman Kent and his colleagues at the Office of National Estimates wrote: ‘Although it is impossible to determine which course the Kremlin is likely to adopt, we believe that […] an attack on Yugoslavia in 1951 should be considered a *serious possibility* [emphasis added]’ [189]. When asked by the chairman of the State Department's Policy Planning Staff to put a number on ‘serious possibility’, Mr Kent asked his colleagues which odds they had in mind. It turned out that the intelligence officers, all expert forecasters, had very different odds in mind, ranging from 20–80 (20%) to 80–20 (80%) in favour of an invasion, rendering the recommendation useless. To avoid such miscommunication, a shared metric for exchanging information must be agreed upon at the outset of group discussions [81,86,173].

### 8.2. How to avoid common traps

#### 8.2.1. Weighting opinions

The risks of equality bias can be countered if group members have equal competence at the task in hand [172]. Failing this, groups could decide to explicitly weight individual contributions [190]. Both are tricky: objective measures of competence are hard to come by and markers of reliability may be misleading.

#### 8.2.2. Anonymous interaction

The risks of social conformity can sometimes be avoided by granting anonymity [191]. For example, the value of anonymous opinions is appreciated in pre-publication reviews. Honest feedback from peers and experienced experts is crucial for science to advance, but is difficult to realize in social situations. An unwanted side effect of anonymity is that it carries the well-known risk of free-riding and self-interest going undetected [145,192].

#### 8.2.3. Uncovering information

There are various techniques which may help groups overcome shared information bias. One of them, *instructed dissent*, is to ask a subset of group members to play devil's advocate [193], always adopting the position opposite to the current consensus, or to ask each group member to adopt a position regardless of their individual stance [194]. A problem, however, is that *contrived* advocacy often has less influence on the listener compared to *genuine* advocacy, possibly because the arguments for an adopted position are made with less confidence [195]. Another technique is to have a *no-interruption rule*; it tends to be unshared ideas, or group members who bring diversity to the discussion, such as women in male-dominated environments, who are cut short [196]. Each group member may also be allocated a fixed amount of floor time as speaking time tends to correlate with factors that are not relevant to the task at hand, such as power and gender [197,198]. Finally, when time permits, long discussions increase the probability that unshared information is eventually brought up [199].

#### 8.2.4. Explicit rules

While free interaction is a good way to search the hypothesis space, it may also lead to a rapid narrowing of ideas; for example, group members may fixate on a small part of the hypothesis space or adapt to each other's ideas too quickly [114,116]. One technique designed to help groups overcome such information loss is the *Delphi method*, developed by members of the RAND Corporation in the 1950s [200]. This method has been shown to lead to better group decisions than unconstrained discussion for a variety of tasks [201]. In its classic form, group members privately outline their individual opinion and rationale; this information is then passed on to a moderator who collates an anonymized summary; group members are presented with this information and given the opportunity to revise their initial opinion; this process is repeated until consensus, or some pre-specified stop criterion, is reached [202]. There are several reasons why the Delphi method works: there is no fixation of the group discussion; anonymity removes the issues of evaluation apprehension and defensive bolstering; there is less room for production blocking as group members do not have to compete for speaking time, nor can they interrupt each other's train of thought; and the iterative process allows for individual changes of mind once good arguments surface [202].

#### 8.2.5. Good leadership

Because of the complexities of group decision-making, it is wise to have a monitoring process in place. This can be achieved through a group chair or leader. The chair should make explicit the precise pitfalls that the decision-making process should avoid. The chair should be aware of the range of biases that might be at play and point them out when they occur. The chair should be particularly sensitive to group members not agreeing on the nature of the problem to be solved. In this case, discussion of their respective solutions will not be fruitful. Experience suggests that, sometimes, different ideas about which course of action is best to take may be rooted in different ideas about the problem at hand. Here, much time can be wasted in arguing about seemingly alternative solutions, which are, in fact, solutions to entirely different problems. Another important role of the group leader is to take into account the long-term goals of the group, especially when these are in conflict with immediate goals. For the overall effectiveness of a group, and the quality of its decisions over time, listening to everyone can be important, even if that means overweighting poor information for specific decisions. This is less the case for one-off panels assembled for a particular purpose, but it is a significant factor in building an effective group to deliver a long-term project. The chair should also be aware of the trade-off between speed and accuracy. Sometimes, the group moves too slowly and loses out on other opportunities. Sometimes, the group moves too quickly and delivers bad decisions as a result.

## 9. Conclusion

Our focus on biases may have given the impression that biases are something that we always need to overcome to make good decisions*.* However, this is not the story that we want to propagate. Biases are the reality of our cognitive system. It is the cost we pay for efficiency. We can think of biases as priors in the Bayesian framework. These priors have been passed on to us partly by nature and partly by culture. They often stand us in good stead. Biases can help us make decisions in novel situations where our learned habits cannot guide us. They avoid dithering, which can be fatal. But, biases are a bad thing when they are out of date and inappropriate. They can also lead us to get stuck on local maxima.

Can we change our biases consciously? We are not usually conscious of our biases at the time we make a decision, but we can reflect on them afterwards and they are malleable. They are also more obvious to others, especially diverse others, than they are to ourselves, and can thus be the subject of discussion. Why should we wish to change our biases? The reason is simple: if we surrendered to our biases wholesale, there would only ever be business as usual, and we would not improve our models of the world and find better solutions to the many problems that we face.

## Figures and Tables

**Figure 6.**
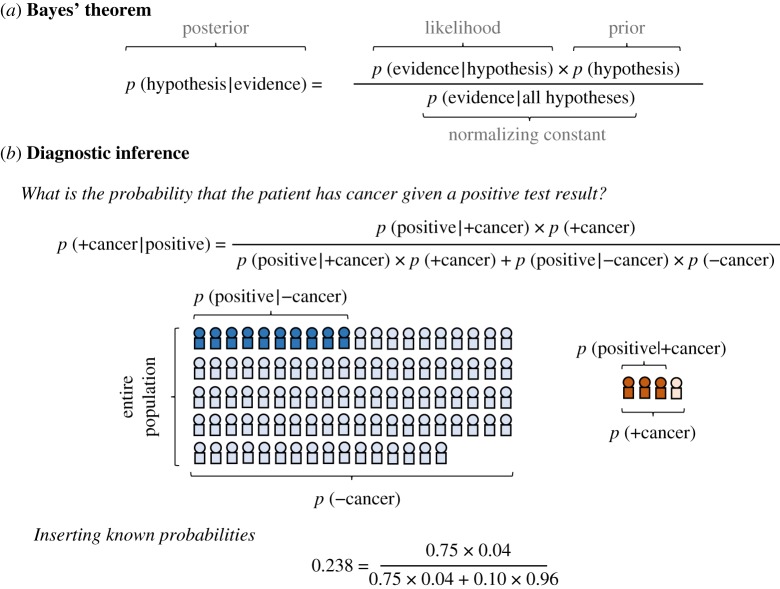
Bayesian inference. (*a*) Bayes' theorem. Here, ‘*p*’ means *probability* and ‘|’ means *given*, so *p*(hypothesis|evidence) means *probability that the hypothesis is true given the evidence*. The *normalizing constant*, which is not discussed in the appendix, is the marginal likelihood of observing the data irrespective of the hypothesis and ensures that the posterior probabilities for the different hypotheses add up to 1. (*b*) Using Bayes' theorem in diagnostic inference. A doctor wishing to compute the probability that a patient has prostate cancer given a positive test result, *p*(+cancer|positive). We know that the test makes correct detections in 75% of cases, *p*(positive|+cancer) = 0.75, and gives false positives in 10% of cases, *p*(positive|−cancer) = 0.10. We also know that the base-rate of prostate cancer is only 4%, *p*(+cancer) = 0.04.

**Figure 7.**
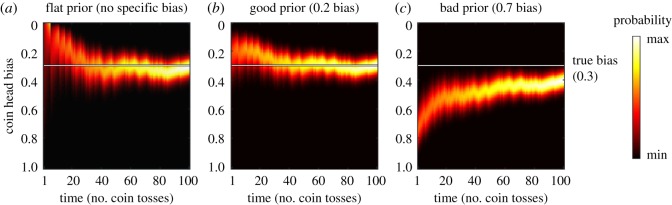
Bayesian updating. We are presented with a biased coin that gives heads on 30% of tosses. Each *heat map* shows how our posterior (colours) over hypotheses about coin bias (vertical axis) evolves as we observe more and more coin tosses (horizontal axis). The white line indicates the true value. The prior at a given point in time is the posterior from the previous point in time. The prior for time 1 was set to be (*a*) flat across hypothesis, (*b*) centred on the truth or (*c*) centred on a wrong value.

**Figure 8.**
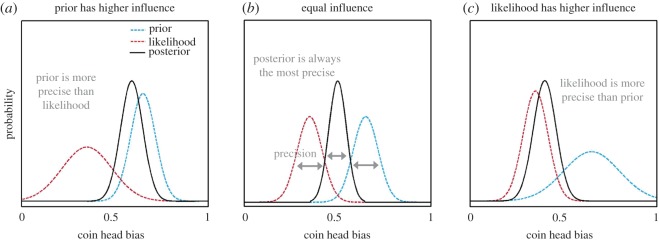
Precision. The plot shows the prior, the likelihood and the posterior represented with probability distributions rather than point estimates (i.e. single numbers). We consider a biased coin as in figure 7. The width of a distribution is known as its *variance* and *precision* is the inverse of variance: 1/variance. When the prior and the likelihood are integrated, the posterior distribution will move towards the more precise component. Note that, because we combine multiple sources of information, the posterior distribution is more precise than either of the two distributions.

## Data accessibility

This article has no supporting data.

## Appendix A. Bayesian inference

Hypotheses play a central role in decision-making: when we make a decision, we typically weigh up the evidence in favour of, and the evidence against, competing hypotheses about the current problem and how best to solve it. For example, by examining a patient's medical history and test results, a doctor seeks to identify the diagnosis that is most likely to be correct and the treatment that is most likely to be successful. Ideally, we wish to use a principled method for evaluating a hypothesis and for updating it as new evidence arrives. Bayesian inference achieves exactly that by leveraging past experience and new evidence in a statistical manner. The fundamental Bayesian idea is that we can represent our degree of belief in a hypothesis as a probability. We can therefore use the laws of probability to update our degree of belief as new evidence comes to light.

The workhorse in Bayesian inference is Bayes' theorem, named after Thomas Bayes (1701–1761). The theorem allows us to ask: what is the probability that a hypothesis is true given the new evidence? There are two major components that enter into this calculation. The first component is the *prior*. The *prior* describes our degree of belief in the hypothesis *before* considering the new evidence; it encapsulates background information and relevant past experience. The second component is the *likelihood*. The likelihood describes how consistent we think the new evidence is with the hypothesis; for example, how likely we think we are to observe heads on 9 out of 10 coin tosses under the hypothesis that the coin is fair. The likelihood critically depends on our *model of how the world works*; for example, we assume that coins fall either way equally often if they are fair. Another term that we need to understand is the *posterior*. This is the integration of the prior and the likelihood. The posterior describes our degree of belief in the hypothesis *after* incorporating the new evidence; it can be used to evaluate the goodness of the hypothesis, and it can function as the prior in the next iteration of Bayes’ rule. We illustrate Bayes' rule and Bayesian updating in figures 6 and 7.

Why is it important that we take into account both the prior and the likelihood? Consider the example in figure 6. A doctor administers a diagnostic test for prostate cancer, which makes correct detections in 75% of cases and gives false positives in only 10%. What is the probability that the patient has prostate cancer given a positive result? Most people, even some doctors, will suggest about 70%, focusing on the result of the diagnostic test [203]. If, however, prostate cancer is only found in 4% of men, then, as the calculation in figure 6 shows, the correct answer is only about 24%. It is also important that we take into account the *precision* of the prior and the likelihood. If the estimate of the base rate of prostate cancer is vague or comes from an unreliable source, then we should give more weight to the diagnostic test. In Bayesian inference, precision can be implemented by representing beliefs with probability distributions rather than single point estimates. We illustrate the concept of precision in figure 8.

## Appendix B. Group performance under different strategies

For all simulations, we assumed that the group and its members are estimating the value of a continuous variable (e.g. the weight of an Ox as in Galton's classic study). We explored the effect of different strategies and circumstances on the precision (reliability) of the joint estimate.

**B.1. Reliability of joint estimate grows with group size**

We assume that the individual estimate, *x*, of each group member, *i*, is sampled from a Gaussian distribution, *N*(*s*, *σ*^2^), where the mean, *s*, is the value of the variable being estimated and where the standard deviation, *σ*, is set to 1. We assume that the group computes its joint estimate by averaging the individual estimates:

$$z=\frac{1}{n}\overset{n}{\underset{i=1}{\sum }}x_{i},$$

where *n* is the group size. Since the errors of individual estimates follow a Gaussian distribution, the error of the joint estimate will do the same. For a given group size, the standard deviation of the joint estimate is

$$\sigma_{\mathrm{group}}=\frac{\sigma }{\sqrt{n}},$$

and the precision of the joint estimate is

$$p_{\mathrm{group}}=\frac{1}{\sigma_{\mathrm{group}}^{2}}.$$

It turns out that there is a linear relationship between group size and reliability.

**B.2. It is important to weight opinions by their reliability**

We assume that the individual estimate, *x*, of each group member, *i*, is sampled from a Gaussian distribution, $N(s,\sigma_{i}^{2})$, where the mean, *s*, is the value of the variable being estimated and where the standard deviation, *σ_i_*, is sampled uniformly from the range 1–4. As such, the estimates of the best group member can have up to 16 times less variance than the worst group member. When the individual estimates differ in terms of their precision, the optimal strategy is to weight each estimate by its precision:

$$z_{\mathrm{optimal}}=\frac{\sum_{i=1}^{n}p_{i}x_{i}}{\sum_{i=1}^{n}p_{i}},$$

where *n* is the group size. Under this optimal strategy, the precision of the joint estimate is the sum of individual precisions:

$$p_{\mathrm{optimal}}=\overset{n}{\underset{i=1}{\sum }}p_{i},$$

where the individual precision is $p_{i}=1/\sigma_{i}^{2}$.

The simple averaging strategy does not take into account individual precision:

$$z=\frac{1}{n}\overset{n}{\underset{i=1}{\sum }}x_{i},$$

where *n* is the group size. For a given group size, the standard deviation of the joint estimate is

$$\sigma_{\mathrm{simple}}^{2}=\frac{\sum_{i=1}^{n}\sigma_{i}^{2}}{n^{2}},$$

and the precision of the joint estimate is

$$p_{\mathrm{simple}}=\frac{1}{\sigma_{\mathrm{simple}}^{2}}.$$

We computed each data point for the figure by averaging across 1000 simulations.

**B.3. Correlated opinions reduce the reliability of the joint estimate**

We assume that the individual estimate, *x*, of each group member, *i*, is sampled from a multivariate Gaussian distribution, N(s,Σ), where **s** is the mean vector

$$\text{s}=\left(\begin{array}{c} s\\ \vdots \\ s \end{array}\right),$$

where *s* is the same for all group members, and where Σ is the covariance matrix in which $\Sigma_{j,j}=1$ and $\Sigma_{j,k}=r$ for i≠j, where *r* describes the correlation between individual estimates. Here, we used *r* = 0.1, *r* = 0.3 and *r* = 0.6. We assumed that the group computes its joint estimate, *z*, by averaging the individual estimates:

$$z=\frac{1}{n}\overset{n}{\underset{i=1}{\sum }}x_{i}.$$

By conducting simulations, we can compute the expected variance under the simple averaging strategy as the squared error of the mean joint estimate:

$$\sigma_{\mathrm{group}}^{2}=\frac{\sum_{l=1}^{q}z_{l}^{2}}{q},$$

where *q* is the number of simulations. We can then compute the expected precision under the simple averaging strategy as

$$p_{\mathrm{group}}=\frac{1}{\sigma_{\mathrm{group}}^{2}}.$$

We computed each data point for the figure by averaging across 1000 simulations.
